# Photoautotrophic and Mixotrophic Cultivation of Polyhydroxyalkanoate-Accumulating Microalgae Consortia Selected under Nitrogen and Phosphate Limitation

**DOI:** 10.3390/molecules26247613

**Published:** 2021-12-15

**Authors:** Parichat Phalanisong, Pensri Plangklang, Alissara Reungsang

**Affiliations:** 1 Research Group for Development of Microbial Hydrogen Production Process from Biomass, Khon Kaen University, Khon Kaen 40002, Thailand; parichat.ppg@gmail.com (P.P.); penspl@kku.ac.th (P.P.); 2 Department of Biotechnology, Faculty of Technology, Khon Kaen University, Khon Kaen 40002, Thailand; 3 Academy of Science, Royal Society of Thailand, Bangkok 10300, Thailand

**Keywords:** third-generation biomass, biodegradable plastic, sugarcane juice, macromolecules

## Abstract

Microalgae consortia were photoautotrophically cultivated in sequencing batch photobioreactors (SBPRs) with an alteration of the normal growth and starvation (nutrient limitation) phases to select consortia capable of polyhydroxyalkanoate (PHA) accumulation. At the steady state of SBPR operation, the obtained microalgae consortia, selected under nitrogen and phosphate limitation, accumulated up to 11.38% and 10.24% of PHA in their biomass, which was identified as poly(3-hydroxybutyrate) (P3HB). Photoautotrophic and mixotrophic batch cultivation of the selected microalgae consortia was conducted to investigate the potential of biomass and PHA production. Sugar source supplementation enhanced the biomass and PHA production, with the highest PHA contents of 10.94 and 6.2%, and cumulative PHA productions of 100 and 130 mg/L, with this being achieved with sugarcane juice under nitrogen and phosphate limitation, respectively. The analysis of other macromolecules during batch cultivation indicated a high content of carbohydrates and lipids under nitrogen limitation, while higher protein contents were detected under phosphate limitation. These results recommended the selected microalgae consortia as potential tools for PHA and bioresource production. The mixed-culture non-sterile cultivation system developed in this study provides valuable information for large-scale microalgal PHA production process development following the biorefinery concept.

## 1. Introduction

The potential of fossil fuel depletion and environmental problems caused by the use of plastics originating from petroleum hydrocarbons has driven the exploration of biodegradable plastics as environmentally friendly substitutes. Polyhydroxyalkanoates (PHAs) are biopolymers that are fully biodegradable in the environment. These polymers are mainly classified into three classes based on the side chains of their monomers, namely, short-chain-length PHAs (scl-PHAs), medium-chain-length PHAs (mcl-PHAs), and long-chain-length PHAs (lcl-PHAs). Scl-PHAs have properties similar to those of petroleum-based conventional thermoplastics, while mcl-PHAs are elastomeric in nature [1,2,3]. Therefore, they have attracted attention from many researchers as alternatives to solve the problems associated with petroleum-based bioplastics. As they are biocompatible, PHAs have recently been applied in the biomedical field as drug delivery carriers, bone and tissue replacement materials, surgical noodles, etc. [4,5,6].

Various microorganisms, such as bacteria, haloarchaea, and microalgae, can accumulate PHAs as carbon and energy storage compounds under stress conditions, such as nitrogen and phosphate deficiencies [7,8,9]. Autotrophic microalgae and cyanobacteria have been proposed to have advantages over heterotrophic bacteria. They represent an exceptionally diverse group of microorganisms that can thrive in freshwater, seawater, and wastewater and are highly tolerant to environmental stress. In addition, they require fewer nutrients for cellular growth and are capable of fixing atmospheric CO_2_, which could lessen the global crisis regarding greenhouse gas emissions [10]. Moreover, microalgae are also known to synthesize a variety of biochemical products, such as proteins, lipids, carbohydrates, and pigments, which can be extracted and converted to value-added products using an integrated biorefinery approach [11,12].

Photoautotrophic PHA production has been reported in diverse strains of cyanobacteria and a few strains of green microalgae, but the PHA biomass content is relatively low compared to that of heterotrophic PHA-producing bacteria [13]. However, PHA accumulation by microalgae can be promoted to satisfactory levels under optimized conditions, such as sufficient carbon and light sources, optimal temperature and pH, and adequate concentrations of nutrients and metal ions in the culture medium. Furthermore, supplementation of organic carbon sources, such as simple sugars and volatile fatty acids, allows the microalgae to grow mixotrophically. Nutrient starvation and/or salt stress conditions were also reported to enhance PHA accumulation by the microalgae [7,13,14]. Successive mixotrophic PHA production has been mainly reported with monoseptic culture of microalgae. Pure culture cultivation requires the supplementation of sterile carbon sources under aseptic conditions to prevent microbial contamination, which makes the large-scale PHA production process economically unfeasible. To overcome this limitation, the development of a mixed culture cultivation system may be of interest for the production of PHAs under septic conditions. In addition, mixed cultures are well adapted to environmental stress and complex carbon sources, allowing the utilization of industrial organic waste streams as low-cost substrates for PHA production [15,16,17,18].

In the present study, mixed cultures of microalgae were photoautotrophically grown with an alteration of the normal growth and starvation (nitrogen or phosphate limitation) phases to select consortia capable of PHA accumulation. Batch accumulation tests were further performed with photoautotrophic and mixotrophic cultivation modes under phosphate and nitrogen limitation to investigate the potential of biomass and PHA production using the selected microalgae consortia. The variation in macromolecules, including proteins, lipids, and carbohydrates, was also examined during PHA production by the selected microalgae consortia.

## 2. Results and Discussion

### 2.1. Enrichment and Selection of PHA-Accumulating Microalgae Consortia

**Figure 1:**
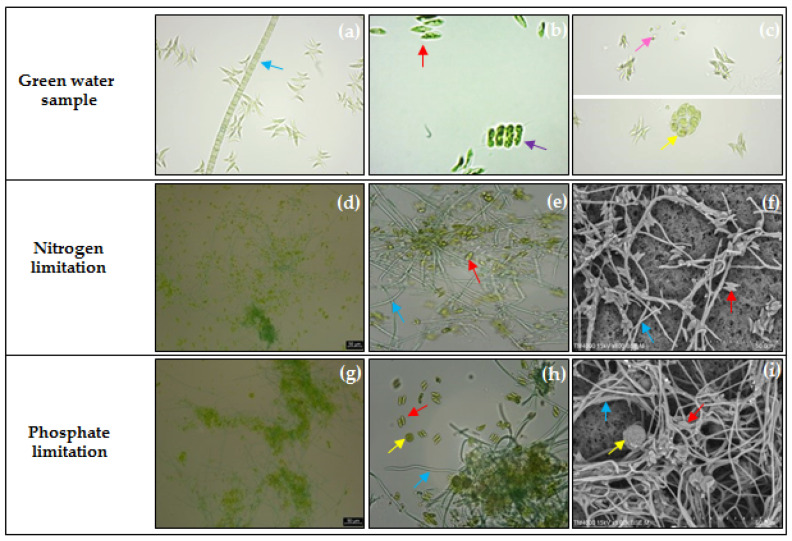
Morphologies of microalgae consortia from green water and the consortia selected under nitrogen and phosphate limitation observed by an optical microscope with 400X magnification (**a**–**c**,**e**,**h**), digital microscope (**d**,**g**), and scanning electron microscope (**f**,**i**). The cells of *Scenedesmus* sp., *Desmodesmus* sp., *Chlorella* sp., *Coelastella* sp., and *Phormidium* sp. are visible as indicated by red, purple, pink, yellow, and blue arrows, respectively.

Microscopic observations indicated that *Scenedesmus* sp., *Desmodesmus* sp., and *Chlorella* sp. were the dominant microalgae in the green water sample, whereas *Coelastella* sp. and *Phormidium* sp. were the minor species (Figure 1). The microalgae consortia obtained after 26 cycles of SBPRs operation were observed under a light microscope and scanning electron microscope (SEM) (Figure 1). *Scenedesmus* sp. and *Phormidium* sp. became the dominant species in the microalgae consortium selected under nitrogen-limited conditions (reactor A), whereas *Phormidium* sp., *Scenedesmus* sp., and *Coelastella* sp. were the dominant species under phosphate-limited conditions (reactor B).

**Figure 2:**
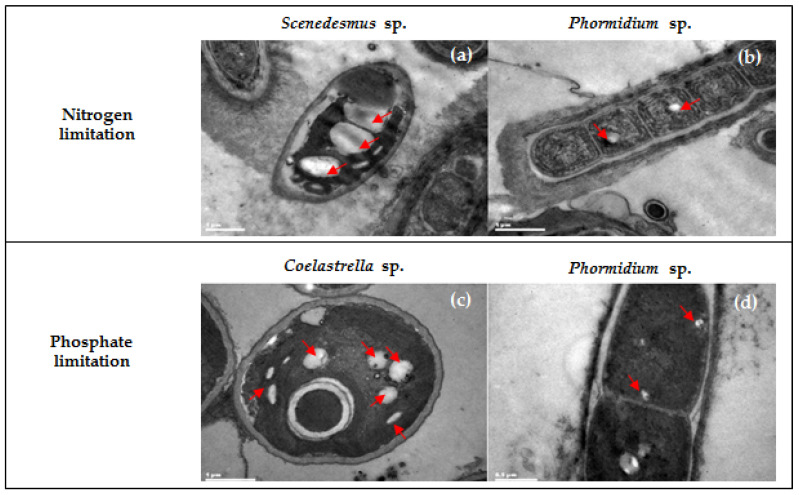
Transmission electron micrographs of dominating microalgae cultivated under nitrogen limitation (**a**,**b**) and phosphorous limitation (**c**,**d**). Red arrows indicate the PHA granules existing in the microalgae cells.

**Figure 3:**
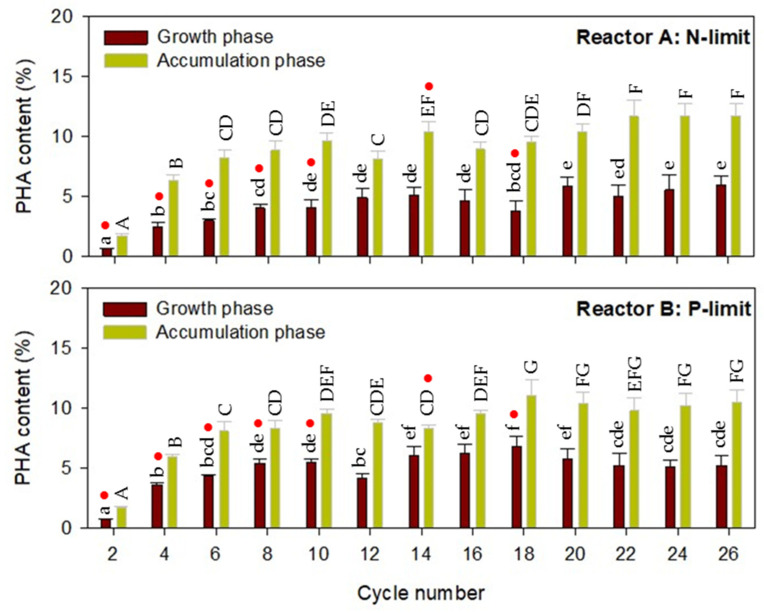
Polyhydroxyalkanoate (PHA) contents of microalgae consortia during photoautotrophic selection in sequencing batch photobioreactors under nitrogen-limited and phosphate-limited conditions. PHA contents observed during the growth phase in different cycle numbers are significantly different if marked with different lower case letters. PHA contents observed during the accumulation phase in different cycle numbers are significantly different if marked with different capital letters (*p* ≤ 0.05, ANOVA/Duncan’s multiple range test). Red dots indicate the significant difference between the data obtained under nitrogen and phosphate limitation (*p* ≤ 0.05, independent-samples *t*-test).

The transmission electron microscopic (TEM) images indicated the presence of insoluble inclusions (white or greyed white granules) in the cytoplasm of the dominant microalgae species (Figure 2). The cells of *Scenedesmus* sp. harbored mainly multiple large granules with an average size of 1.00 μm (Figure 2a). Both small and large granules with diameters ranging between 0.25 and 0.50 μm were observed in *Coelastella* sp. cells (Figure 2c). For *Phormidium* sp. cells, only small granules (0.10–0.25 μm) were observed. In particular, a single granule was found under nitrogen limitation (Figure 3b), while multiple granules were noticed under phosphate limitation (Figure 2d).

To verify the accumulation of PHA by the selected consortia, the microalgae biomass was extracted and analyzed for its PHA content during the selection process in SBPRs. A PHA content of approximately 1.2% was detected in the seed microalgae consortia and gradually increased with the cultivation cycle (until the 20th cycle for reactor A and the 18th cycle for reactor B), noting that higher values were observed at the end of the accumulation phase as compared to those observed at the end of the growth phase (Figure 3). The PHA contents of the microalgae consortia were stable during cycles 20 to 26 of the SBPR operation with an average PHA content of 11.38 ± 0.64% and 10.24 ± 0.32% of cell dry mass after selection under nitrogen limitation (reactor A) and phosphate limitation (reactor B), respectively. Nitrogen and phosphorus are essential nutrients for microalgae biomass production. The limitation of these nutrients is the typical approach applied to induce PHA accumulation in various strains of microalgae [7,14,19,20]. Under nitrogen- and/or phosphate-limiting conditions, the protein synthesis rate and electron transfer activity decrease, hence lowering the total ATP pool while maintaining the activity of NADP reduction to NADPH. Accumulation of intracellular NADPH inhibits citrate synthase, resulting in an increased acetyl-CoA pool, which favors PHA synthesis and consequently increases the PHA content of the cells [7].

**Figure 4:**
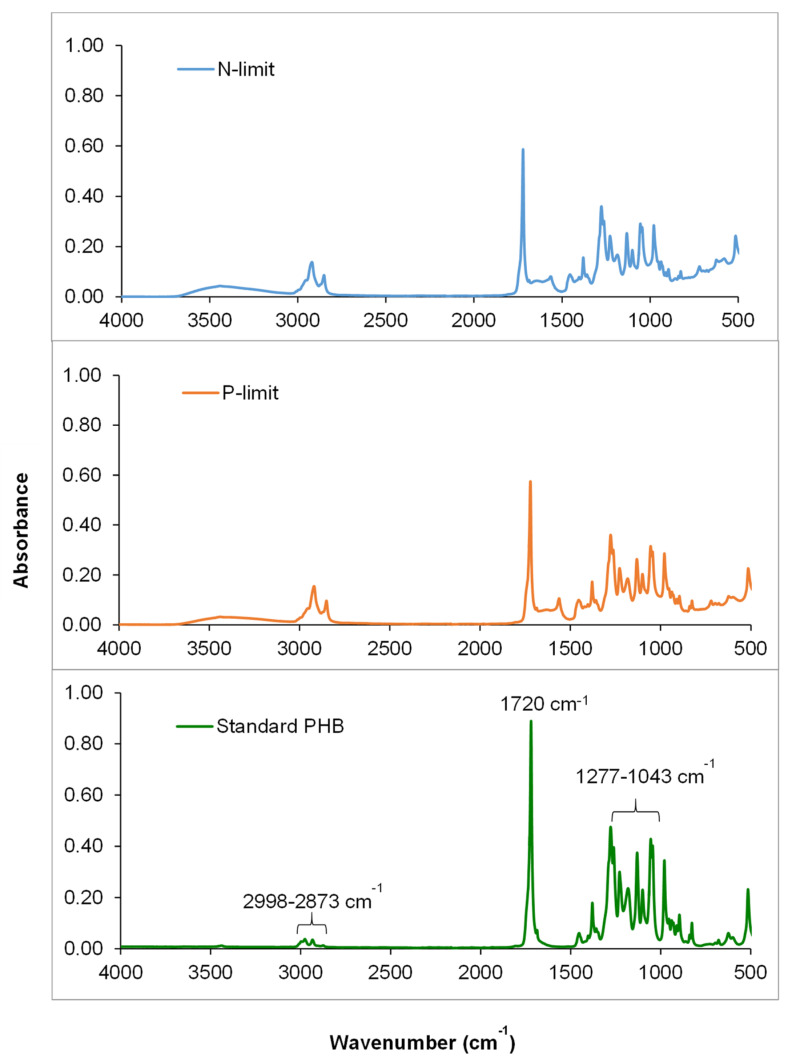
Fourier transform infrared spectra of the extracted PHA from microalgae consortia cultivated under nitrogen and phosphate limitation and standard poly(3-hydroxybutyrate) (P3HB).

Characteristic Fourier transform infrared (FTIR) peaks for the produced PHA and standard P3HB are presented in Figure 4. The FTIR spectra presented noticeable peaks at 1720 and 1175 cm^−1^ denoting the carbonyl group (C=O) of the ester bond and stretching vibration of asymmetric C-O-C, respectively, which are characteristic of PHB functional groups. The CH stretching vibrations of -CH, -CH_2_, and -CH_3_ groups were observed at 2998–2875, 1453, and 1380 cm^−1^, respectively, while the C-O stretching vibration was observed at 1460 cm^−1^. The peaks obtained from the produced PHA were similar to those of standard P3HB, confirming that the polymer produced by the mixed culture of microalgae under nitrogen and phosphate limitation is P3HB. These identical peaks denoting the PHB functional groups were also detected in *Scenedesmus* sp. [7] and *Nostoc muscorum* NCCU-442 [14]. Very limited information has been reported on PHA accumulation by green microalgae. The accumulation of PHA by *Coelastrella* sp. has not yet been reported. *Scenedesmus* sp. was reported as a PHA producer in the study by García et al. [7]. This strain could accumulate approximately 30% of PHA when 1 g/L glucose was used as the carbon source under phosphate limitation. Various strains of cyanobacteria, such as *Phormidium* sp., *N. muscorum*, *Gleocapsa gelatinosa*, *Synechocystis* sp., and *Spirulina platensis*, have been reported to accumulate PHA at 0.51 to 6.44% in photoautotrophic cultivation mode. In addition, a higher PHA content in *N. muscorum* (26.37%) could be achieved under phosphate deficiency by supplementation of glucose (4 g/L) and sodium chloride (0.1 g/L) with pH, temperature, and light/dark alteration optimization [14].

### 2.2. Batch Cultivation of the Selected Microalgae Consortia

#### 2.2.1. Biomass Production and Sugar Utilization

Photoautotrophic (with CO_2_ as a C-source) and mixotrophic (with CO_2_ and sugar as C-sources) batch cultivation of the selected microalgae consortia was conducted to investigate the potential of biomass and PHA production. In all treatments, the microalgae consortia were able to grow without a lag phase. Under the nitrogen-limited conditions, the cumulative biomass production (in grams of dry mass per liter of culture) rapidly increased during the initial phase (0–48 h) of cultivation, and the biomass increased continuously with the lower growth rate thereafter (Figure 4). The microalgae consortium comprised exponentially growing cells from the preculture, and the intracellular nitrogen probably served as the nitrogen source for their growth during the initial phase of cultivation. The slow growth rates detected after 96 h of cultivation represented the normal growth of the microalgae with nitrogen deficiency.

**Figure 5:**
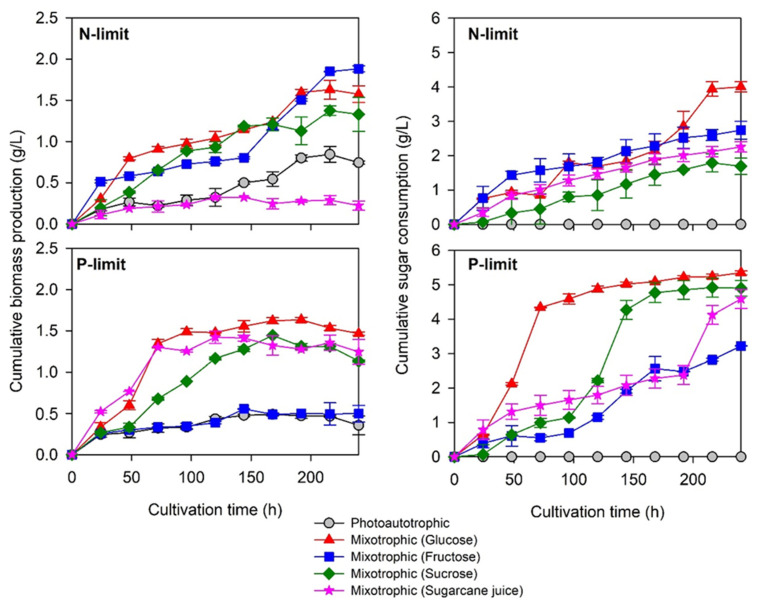
Cumulative biomass production (in grams of dry mass per liter of culture) and cumulative sugar consumption during the batch accumulation test in nitrogen- and phosphate-limiting media in photoautotrophic and mixotrophic cultivation modes.

All the sugar sources were consumed without the lag period except for sucrose, which started to be consumed after 48 h of cultivation (Figure 5). This might be because sucrose is a disaccharide that needs to be hydrolyzed to its monosaccharide components, glucose and fructose, prior to microbial assimilation. At 0 to 48 h of cultivation, biomass production increased, without sucrose consumption, at the same rate as that observed in photoautotrophic cultivation, implying that the microalgae consortia grew autotrophically when the sugar source was hardly assimilated. The enhanced biomass production in the presence of other sugar sources (Figure 5) suggested that both sugar and CO_2_ can be used as carbon sources by the flow of the pentose phosphate and Calvin cycle pathways, which are the main characteristics of mixotrophic cultivation. This combined cultivation mode has the advantage of reducing the inhibition of cell growth induced by the limitation of carbon sources or light [21,22].

By employing photoautotrophic cultivation, the microalgae consortia grew slowly and reached the maximum cumulative biomass production value of 0.844 g/L at 216 h under nitrogen-limited conditions and 0.489 g/L at 168 h under phosphate-limited conditions (Figure 5). In the mixotrophic cultivation mode, the microalgae consortia grew rapidly. The cumulative biomass production increased with the increased cumulative sugar consumption, except for the treatment with sugarcane juice under nitrogen-limited conditions and the treatment with fructose under phosphate-limited conditions (Figure 5). Phosphate-limited conditions led to a faster-growing culture that reached the stationary phase earlier than that under nitrogen-limited conditions, indicating that nitrogen supplementation could promote the rate of sugar consumption and biomass production under the limitation of phosphate. After 240 h of cultivation, among the various carbon sources fed under nitrogen-limited conditions, fructose produced the greatest cumulative biomass production of 1.882 g/L with 4 g/L of sugar utilization, while glucose, sucrose, and sugarcane juice yielded the lower biomass production and sugar utilization efficiencies. Under phosphate-limited conditions, glucose, sugarcane juice, and fructose yielded relatively high cumulative biomass production values ranging between 1.334 and 1.466 g/L with 4.18–5.35 g/L sugar utilization, while fructose gave the lowest cumulative biomass production (0.367 g/L with 3.2 g/L sugar utilization). Although relatively high amounts of sugars (2.25–3.22 g/L) were consumed, low cumulative biomass production was observed (0.323–0.366 g/L) with the presence of sugarcane juice under nitrogen limitation and fructose under phosphate limitation. This result implies that the microalgae consortia might convert sugars to extracellular products under these conditions. Examples of extracellular products obtained during microalgae cultivation are phytohormones, lactic acid, extracellular lipids, and fatty acids [23].

#### 2.2.2. PHA Accumulation

**Figure 6:**
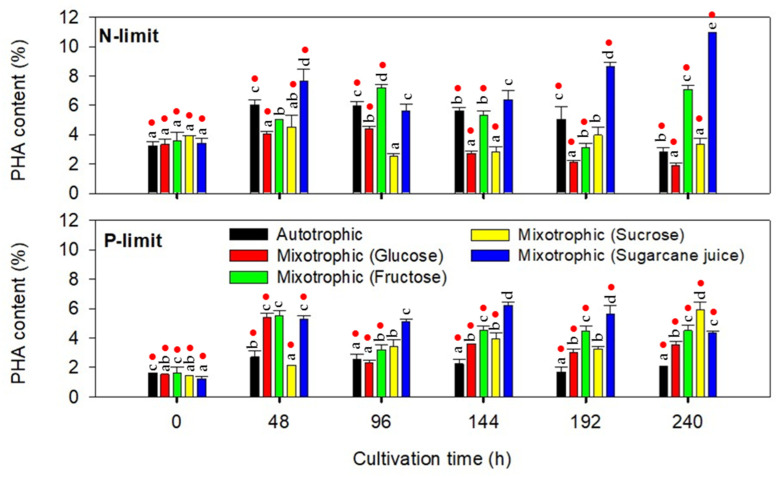
Polyhydroxyalkanoate (PHA) contents of microalgae consortia during the batch accumulation test in nitrogen- and phosphate-limiting media under photoautotrophic and mixotrophic conditions. PHA contents observed in different treatments at the same cultivation time are significantly different if marked with different lower case letters (*p* ≤ 0.05, ANOVA/Duncan’s multiple range test). Red dots indicate the significant difference between the data obtained under nitrogen and phosphate limitation (*p* ≤ 0.05, independent-samples *t*-test).

Microalgae consortia were pre-cultured in nutrient-rich medium under photoautotrophic conditions for four days, with average PHA contents of 3.49% and 1.50% under nitrogen- and phosphate-limited conditions, respectively. Under nitrogen limitation, the average PHA content of the microalgae consortium ranged from 1.92% to 10.94%. The PHA content increased to reach maximum values at 48 or 96 h of cultivation and tended to decrease thereafter. This phenomenon was not observed for the treatment with sugarcane juice, in which the PHA content increased continuously and reached the maximum value at the end of cultivation (Figure 6). According to the biomass production profiles (Figure 5), the maximum PHA content (Figure 6) was achieved after the microalgae exhibited the slow growth rates induced by nitrogen deficiency. The results indicated that nitrogen limitation promoted PHA production in both photoautotrophic and mixotrophic cultivation. However, prolonged nitrogen limitation might limit microbial metabolic processes, such as nucleic acid, protein, and enzyme synthesis, and PHA might be utilized for cell functioning, which indirectly decreases PHA production. In addition, the carbon flux could be driven to the pathways competing for common substrates, such as acetyl-CoA in fatty acid and lipid biosynthesis [24]. Photoautotrophic cultivation and mixotrophic cultivation with glucose, fructose, sucrose, and sugarcane juice supplementation yielded maximum cumulative PHA production of 62, 55, 94, 37, and 100 mg/L, respectively. The results indicated that, under nitrogen limitation, mixotrophic growth with fructose or sugarcane juice supplementation enhanced PHA production by the microalgae consortium as compared to photoautotrophic cultivation. Supplementation with glucose or sucrose could contribute to biomass production rather than PHA accumulation. Supplementing low amounts of glucose has been reported to enhance PHA production by cyanobacteria [14] and *Scenedesmus* sp. [7]. However, García et al. [7] reported a prolonged exponential growth phase, which possibly delayed the PHA biosynthesis phase by supplementing with a high amount of glucose. A high PHA content achieved with low biomass production, with the presence of sugarcane juice, would facilitate the design of two-step mixotrophic biomass production and PHA accumulation by the selected microalgae consortium. Other sugar sources can be supplied during the first step of biomass production to obtain a high concentration of microalgae cells, which can be consequently grown in sugarcane juice-supplemented medium for PHA production.

The PHA accumulation behavior of the microalgae consortium grown under phosphate limitation was different from that observed under nitrogen limitation. The PHA content ranged from 1.24% to 6.2% during the batch accumulation test (Figure 6). The PHA content increased after 48 h of photoautotrophic cultivation and remained stable thereafter. Treatments with glucose and fructose achieved the maximum PHA content during exponential growth, while the treatments with sucrose and sugarcane juice achieved the maximum measurement during the stationary phase. Compared to nitrogen-limited conditions, phosphate limitation led to a lower PHA content, suggesting that nitrogen limitation is a more efficient strategy to enhance PHA accumulation by the selected microalgae consortia in both photoautotrophic and mixotrophic cultivation modes. Photoautotrophic cultivation under nitrogen limitation yielded a maximum cumulative PHA production of 16 mg/L. All the supplemented sugar sources promoted PHA production by the selected microalgae consortium in which the addition of glucose, fructose, sucrose, and sugarcane juice resulted in maximum cumulative PHA production of 74, 48, 103, and 130 mg/L, respectively. The results under phosphate limitation indicated that sugarcane juice and sucrose can be used as organic carbon sources to improve biomass and PHA production by the selected microalgae consortium. The relatively high PHA content achieved during the exponential growth phase in the presence of glucose, sucrose, and sugarcane juice allowed the production of PHA in a one-step mixotrophic cultivation process.

Overall, the results indicated the potential of PHA production by mixed cultures of microalgae under nitrogen and phosphate limitation in both photoautotrophic and mixotrophic cultivation modes. However, the processes need to be improved because the obtained PHA contents and PHA concentrations are still low under the investigated conditions. Nutrient deficiency was reported to enhance PHA accumulation by green microalgae and cyanobacteria, and the performance would be improved by optimizing other environmental factors, such as sugar concentrations, carbon to nitrogen ratios, pH, salt stress, light intensities and wavelengths, light/dark alteration, and trace element supplementation. By applying a light/dark alteration period of 12/12 h, *N. moscorum* accumulated PHA at 20.52%, 11.81%, and 11.46% with the supplementation of 4 g/L of glucose, fructose, and sucrose, respectively, and cultivation was conducted under optimal pH and temperature, phosphate deficiency, and NaCl addition [14]. In a study by García et al. [7], continuous illumination was applied to PHA production by *Scenedesmus* sp. with glucose and ambient CO_2_ as C sources. A PHA content of 29.92% was achieved under phosphate deficiency with 1 g/L glucose and 0.21 mM iron supplementation, while nitrogen limitation yielded a lower PHA content in the range of 9.08–11.68%.

Although optimization of the microalgal PHA production can be achieved at the laboratory scale, the optimization of production, harvesting, extraction, and purification processes at a commercial scale is still challenging, particularly in the economic context. Applying open raceway ponds and using waste streams (wastewater and industrial exhaust gas) as nutrient and carbon sources has been proposed as a cost-effective approach for large-scale microalgae production. However, suitable sites where nearby substrate sources and infrastructure, have suitable climate conditions, and do not compete with arable land need to be identified to locate large-scale production systems [25,26]. Improving the flocculation and filtration methods for microalgae harvesting and identifying low-cost, environmentally friendly, and reusable solvents for PHA extraction would make the process economically feasible. In addition, implementing the biorefinery approach by recovering the co-products in microalgae biomass would be a great solution for improving the economics of PHA production [27]. However, the cost of co-product recovery processes should be minimized to achieve economically commercial-scale PHA production.

#### 2.2.3. Protein, Lipid, and Carbohydrate Production

**Figure 7:**
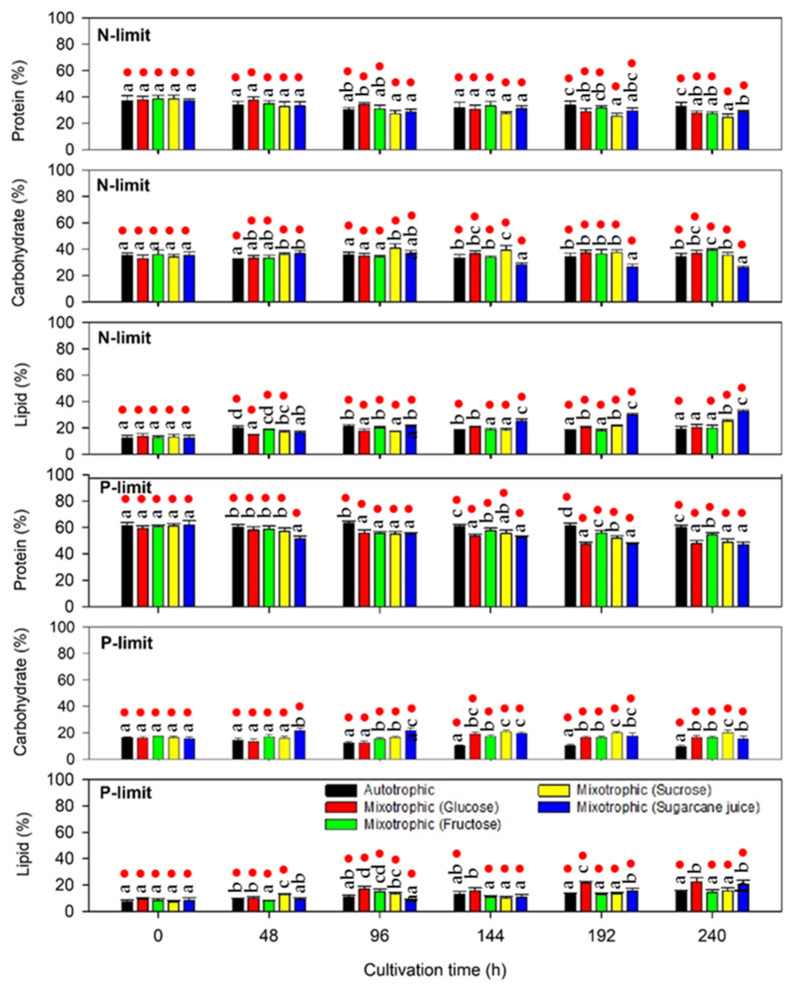
Protein, lipid, and carbohydrate contents of microalgae consortia during batch accumulation test in nitrogen- and phosphate-limiting media under photoautotrophic and mixotrophic conditions. Protein, lipid, and carbohydrate contents observed in different treatments at the same cultivation time are significantly different if marked with different lower case letters (*p* ≤ 0.05, ANOVA/Duncan’s multiple range test). Red dots indicate the significant difference between the data obtained under nitrogen and phosphate limitation (*p* ≤ 0.05, independent-samples *t*-test).

In addition to PHA accumulation, variations in macromolecules, including proteins, lipids, and carbohydrates, during microalgae consortia cultivation were investigated (Figure 7). Higher protein contents (46.87–63.10%) were detected in the microalgae consortium selected under phosphate limitation in comparison to those observed in the microalgae consortium selected under nitrogen limitation (24.58–38.42%). A significant decrease in protein content with cultivation time was observed under nitrogen-deficient conditions, while no obvious change in protein content was observed under phosphate deficiency, except for the treatment with sugarcane juice. The results indicated that protein synthesis by microalgae consortia is mainly influenced by nitrogen rather than phosphate. Proteins are the main biochemical pool of cellular nitrogen; therefore, the presence of available nitrogen in the culture medium typically has a high impact on microbial protein synthesis [28]. However, the response of microalgae to nutrient starvation varies depending on the microalgal species. The carbohydrate content of the microalgae consortia tended to be stable or increased during cultivation, except for the treatment with sugarcane juice, in which an obvious decrease in the carbohydrate content was observed after 144 h of cultivation with both nitrogen and phosphate limitation. A decrease in the carbohydrate content coincided with an increase in the PHA and lipid contents in the presence of sugarcane juice as a carbon source. Previously published reports indicated that the degradation of starch, the main carbohydrate storage compound of microalgae, is concomitant with lipid accumulation [29]. Glucose obtained from the degradation of glycogen, the main cellular carbohydrate storage compound of cyanobacteria, was reported as the key precursor for PHA synthesis, suggesting that the metabolic pathways for carbohydrate and PHA synthesis are interconnected [30,31]. Since acetyl-CoA is the precursor for both PHA and lipid production, their metabolic pathways might compete with each other for their synthesis substrates [7,24]. The results indicated that the selected microalgae consortia can produce various types of macromolecules, and that the biorefinery of their biomass to produce a variety of value-added products together with PHA might be feasible and cost-efficient for industrial-scale production. Microalgal protein, used as a source for peptide or amino acid production, can be used as an animal feed supplement. Carbohydrates can be utilized as substrates by microorganisms to produce market value products, such as bioethanol, while the lipid portion can serve as the raw material for biodiesel production.

## 3. Materials and Methods

### 3.1. Media Preparation

Modified Bold’s basal media were used for microalgae cultivation. Their compositions are presented in Table 1. Bold’s basal medium (BBM) rich in nitrogen and phosphorus (2NBBM) was used for microalgae cultivation in the growth phase, while nitrogen-depleted BBM (named N-limited BBM) and phosphate depleted BBM (named P-limited BBM) were used in the accumulation phase. The media were sterilized in an autoclave at 110 °C for 40 min before being used for microalgae cultivation.

### 3.2. Enrichment and Selection of PHA-Accumulating Microalgae Consortia

A green water sample collected from a freshwater fishpond in Nakhon Ratchasima province, Thailand, was used as the microalgae source in this experiment. Observation and counting under the microscope showed that the green water sample was dominated by *Scenedesmus* sp., *Desmodesmus* sp., and *Chlorella* sp. with proportions of approximately 70%, 15%, and 10%, respectively. *Phormidium* sp. and *Coelastella* sp. were found as minor species with proportions of approximately 3% and 2%, respectively. The microalgae cells were harvested by centrifuging 1 L of the green water sample at 7000 rpm for 10 min using a Sigma 2-16P centrifuge. The cell pellets were rinsed twice with distilled water and used as the seed inoculum for enrichment and selection of PHA-accumulating microalgae consortia.

Enrichment of microalgae consortia was performed in a 2 L laboratory glass bottle, used as a photo-bioreactor (PBR), containing 1.8 L 2NBBM under 6000 lx illumination from cool white fluorescent lamps, and a 12/12 h dark/light cycle. The reactor contents were continuously mixed using a magnetic stirrer. During the light period, CO_2_ (10% in air) was supplied as a carbon source at 0.2 vvm, while air without added CO_2_ was supplied during the dark period. The PBR was operated at 26 ± 3 °C in a temperature-controlled room for four days. The microalgae cells were then harvested by centrifugation, and the resulting microalgae pellets were rinsed and used as inoculum to select PHA-accumulating microalgae consortia.

For the selection process, photoautotrophic cultivation with a nitrogen- (reactor A) or phosphate- (reactor B) limiting period was performed in 2 L sequencing batch photobioreactors (SBPRs) (1.8 L working volume) with biological duplicates. An initial microalgae cell concentration of 0.20 ± 0.012 g/L was applied. The SBPRs were operated at 26 ± 3 °C in a temperature-controlled room. The operating cycle period was set at 96 h, consisting of a 48 h growth phase and 48 h PHA accumulation phase. The cycle structure for the growth phase comprised an initial period of 2NBBM feeding (10 min; 1530 mL), a reaction period for the growth phase (42 h), a settling period (5 h), a withdrawal period (10 min; 1530 mL), and an idle period (10 min). The cycle structure for the PHA accumulation phase comprised a period of N-limited BBM (reactor A) or P-limited BBM (reactor B) feeding (10 min; 1530 mL), a reaction period for the PHA accumulation phase (42 h), a settling period (5 h), a withdrawal period (10 min; 1530 mL), and an idle period (10 min). During both reaction periods, the SBPRs were illuminated with cool white fluorescent light at 6000 lx, and the reactor contents were mixed using a magnetic stirrer. CO_2_ (10% in air) was supplied at 0.2 vvm as a carbon source during the reaction phase of PHA accumulation. During the reaction phase of microalgae growth, air without added CO_2_ was supplied, in which a small amount of CO_2_ was naturally present, and the intracellular accumulating macromolecules were utilized as the carbon source for growth. In each SBPR cycle, the culture broth was taken at the end of the reaction period of growth and accumulation phases to determine the biomass concentration, PHA concentration, and PHA content. Every two cycles, 50% of the culture broth was removed without the settling period, and the same volume of fresh medium was added to the reactors to avoid self-shedding at a high density. The SBPRs were operated until the PHA content in the biomass was stable (less than 5% variation) to select the microalgae consortia capable of PHA accumulation. The morphology of the microalgae cells was observed using a light microscope (Olympus C011) and SEM (Hitachi, TM4000Plus). The microalgae species were morphologically identified according to relevant keys suggested by Goecke et al. [32], Hegewald [33], and Hašler et al. [34]. Transmission electron microscopy (TEM) (Philips, TECNAI 20) was used to observe the PHA granules inside the microalgae cells. PHA was extracted from the microalgae consortia using a dispersion technique modified from the method of Hahn et al. [35]. Briefly, the dried biomass (50 ± 5 mg) was treated with dispersions of 5 mL of chloroform and 5 mL of 5% hypochlorite solution at 30 °C for 1 h. The dispersion was then centrifuged, and the bottom phase (chloroform containing PHAs) was filtered and recovered using non-solvent precipitation and filtration. A mixture of methanol and water (7:3 *v*/*v*) was used for non-solvent precipitation in five volumes of chloroform. The functional groups of the PHA pellets were analyzed using FTIR spectroscopy.

### 3.3. Batch Accumulation Test with Photoautotrophic and Mixotrophic Cultivation

**Figure 8:**
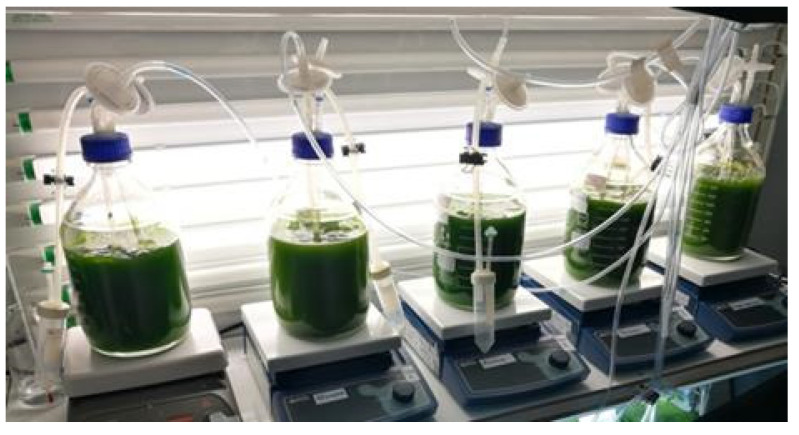
Photobioreactor for the batch accumulation test.

The accumulation performance of the microalgae consortia selected in reactors A and B was investigated by conducting batch cultivation in a 1.8 L working volume PBR (Figure 8) using the same procedures of temperature control, light illumination, mixing, and CO_2_ and air supply as described for PBR operation in Section 2.2. Photoautotrophic and mixotrophic cultivations under nitrogen-limited and phosphate-limited conditions were tested in eight experimental runs with biological duplicates (Table 2). Each PBR experiment was started up by cultivating the seed inoculum, which was obtained from reactor A or B in 2NBBM (growth phase), for four days in which the biomass concentrations ranged from 1.1–1.5 g/L. All the culture broth was withdrawn from each PBR, and the microalgae biomass was harvested by centrifugation and washed twice with sterile distilled water to remove residual nutrients. The obtained biomass was reinoculated into the PBR containing N-limited BBM or P-limited BBM (accumulation phase), according to the experimental design (Table 2). Glucose, fructose, sucrose, or sugarcane juice at a total sugar concentration of approximately 5 g/L was added into the N-limited BBM or P-limited BBM as the organic carbon source in the mixotrophic cultivation tests (Table 2). CO_2_ (10% in air) was supplied as an inorganic carbon source for both mixotrophic and photoautotrophic cultivations. The culture broth was collected every 24 h for the total sugar concentration [36], biomass concentration, and biomass composition (PHA, carbohydrate, lipid, and protein contents of the biomass) analyses. For biomass concentration analysis, the culture broth was centrifuged, and the cell pellets were washed with distilled water prior to oven drying at 80 °C until a constant weight was obtained. The biomass dry weight was then determined and expressed as g/L. PHA was extracted from the microalgae biomass by dispersion following the method described by Hahn et al. [35]. The total carbohydrate and protein contents of the biomass were analyzed according to the method described by Pruvost et al. [37], and the lipid content was analyzed following the methods of Mishra et al. [38].

### 3.4. Statistical Analysis

All bioreactor experiments and analytical measurements were performed in duplicates. The values presented in the figures and tables present the mean ± SD. The effects of carbon sources supplied for microalgae cultivation were analyzed using analysis of variance (ANOVA) followed by Duncan’s multiple range test. The data from the nitrogen- and phosphate-limiting conditions were compared using the independent samples *t*-test. SPSS program version 17.0 (SPSS Inc., Chicago, IL, USA) was used for the statistical analysis. A *p*-value of less than or equal to 0.05 was considered statistically significant.

## 4. Conclusions

Microalgae consortia capable of accumulating PHAs under nitrogen- and phosphate-deficient conditions were obtained, accumulating up to 11.38% and 10.24%, respectively, of PHA in their biomass, which was identified using FTIR as P3HB. Batch accumulation tests using the obtained microalgae consortia revealed that sugar source supplementation enhanced biomass and PHA production, while the highest PHA content and cumulative PHA production were achieved with sugarcane juice. Carbohydrates and lipids were observed as concomitant products along with PHA production. This work recommends the selected microalgae consortia as a potential tool for PHA and bioresource production, which would be valuable information for large-scale production process development following the biorefinery concept. Sugarcane juice can be used as a low-cost and locally available carbon source to promote mixotrophic PHA accumulation by the microalgae consortia. However, the low biomass production under nitrogen limitation in the presence of sugarcane juice should be solved by exploring two-step biomass production and PHA accumulation to achieve high PHA production. The effects of environmental conditions, such as sugar and nutrient concentrations, light intensity and wavelength, and light/dark alteration, should be further investigated to improve PHA production by the selected microalgae consortia.

## Figures and Tables

**Table 1:** Composition of modified Bold’s basal media used in this study.

Components	Concentration of Stock Solution (g·L^−1^)	Volume of Stock Solution (mL/L)		
		2NBBM	N-Limited BBM	P-Limited BBM
(1) NaNO_3_	25	20	None	20
(2) CaCl_2_·2H_2_O	2.5	10	10	10
(3) MgSO_4_·7H_2_O	7.5	10	10	10
(4) K_2_HPO_4_	7.5	8	8	None
(5) KH_2_PO_4_	17.5	8	8	None
(6) NaCl	2.5	10	10	10
(7) FeSO_4_·7H_2_O	4.98	1	1	1
(8) H_3_B_3_	11.42	1	1	1
(9) Alkaline EDTA solution		1	1	1
(9.1) EDTANa_2_	50			
(9.2) KOH	31			
(10) Trace elements solution		1	1	1
(10.1) ZnSO_4_·7H_2_O	8.82			
(10.2) MnCl_2_·4H_2_O	1.44			
(10.3) MoO_3_	0.71			
(10.4) CuSO_4_·5H_2_O	1.57			
(10.5) Co(NO_3_)_2_·6H_2_O	0.49			

**Table 2:** Batch accumulation test with photoautotrophic and mixotrophic cultivation.

Treatment	Source of Inoculum *	Growth Phase		PHA Accumulation Phase	
		Medium	C-Source	Medium	C-Source
Auto-N	Reactor A	2NBBM	CO_2_	N-limited BBM	CO_2_
Mixo-N-Glu	Reactor A	2NBBM	CO_2_	N-limited BBM	Glucose/CO_2_
Mixo-N-Flu	Reactor A	2NBBM	CO_2_	N-limited BBM	Fructose/CO_2_
Mixo-N-Suc	Reactor A	2NBBM	CO_2_	N-limited BBM	Sucrose/CO_2_
Mixo-N-SCJ	Reactor A	2NBBM	CO_2_	N-limited BBM	Sugarcane juice/CO_2_
Auto-P	Reactor B	2NBBM	CO_2_	P-limited BBM	CO_2_
Mixo-P-Glu	Reactor B	2NBBM	CO_2_	P-limited BBM	Glucose/CO_2_
Mixo-P-Flu	Reactor B	2NBBM	CO_2_	P-limited BBM	Fructose/CO_2_
Mixo-P-Suc	Reactor B	2NBBM	CO_2_	P-limited BBM	Sucrose/CO_2_
Mixo-P-SCJ	Reactor B	2NBBM	CO_2_	P-limited BBM	Sugarcane juice/CO_2_

* Seed inocula were obtained from the microalgae selection under nitrogen-limited (Reactor A) or phosphate-limited (Reactor B) conditions.

## Data Availability

The data presented in this study are available in this article.

## Sample Availability

Not available.
